# Point-of-care HIV RNA testing and immediate antiretroviral therapy initiation in young adults seeking out-patient care in Kenya

**DOI:** 10.1097/QAD.0000000000002110

**Published:** 2019-03-14

**Authors:** Eduard J. Sanders, Oscar Chirro, Clifford Oduor, Juma Mangi, Elizabeth Wahome, Matthew A. Price, Hubert C. Gelderblom, Elisabeth M. van der Elst, Susan M. Graham

**Affiliations:** a Kenya Medical Research Institute-Wellcome Trust Research Programme, Kilifi, Kenya; b Nuffield Department of Medicine, University of Oxford, UK; c Malindi Sub-County Hospital, Malindi, Kenya; d International AIDS Vaccine Initiative, New York, New York, USA; e University of Washington, Seattle, Washington, USA.

## Abstract

We evaluated outcomes of an HIV-1-testing intervention using rapid HIV tests followed by point-of-care *Xpert Qual* testing for HIV-1 RNA. Of 706 young urgent-care seeking participants evaluated, 24 (3.4%) had chronic HIV (antibody-positive), 3 (0.4%) acute HIV-1 (*Qual-*positive, antibody-negative), and 3 (0.4%) early HIV-1 infection (*Qual-*positive, antibody-discordant). Overall, 21 (70.0%) diagnosed patients started antiretroviral therapy after a median of 4 days (range 0–71). HIV-1 RNA testing led to an increase in confirmed diagnoses by 25%.

Many adults with acute HIV-1 infection (AHI) seek care for symptoms, whether at emergency departments in well resourced settings [1], or in outpatient clinics in resource-constrained settings [2]. Identifying these individuals is an important public health strategy that could limit HIV-1 transmission, provided they can be diagnosed [2], linked to care [3], and start immediate antiretroviral therapy (ART), as recommended by the WHO [4]. Unfortunately, AHI detection is not supported by policy in sub-Saharan Africa, where testing strategies focus exclusively on identifying adults with undiagnosed prevalent infection.

Acute HIV-1 infection is the phase of HIV-1 infection immediately after acquisition, during which 40–90% of patients develop symptoms [5]. During AHI, anti-HIV antibodies are undetectable, but HIV-1 RNA and p24 antigen are present. Once HIV antibodies develop, the phase is usually referred to as early HIV infection (EHI) [6]. Symptoms develop around 2 weeks after HIV-1 acquisition, just preceding the peak in viral load [7,8]. AHI symptoms are more common in patients infected with subtype A than subtype C or D [9]. In Kenya, where most infections are subtype A, we have found that most AHI patients (69%) seek care for symptoms [10].

In 2015, we developed a consensus risk score to target AHI screening using pooled cohort data from Kenya, Malawi, and South Africa [11]. The consensus risk score had 90.0% sensitivity and 74.1% specificity to identify AHI among MSM followed in a cohort study in Kenya, and reduced the proportion of MSM needing testing to 26.1% of the cohort [11]. The yield of this algorithm among general outpatients in Kenya is unknown.

In the present study, we assessed the outcomes of an HIV-testing intervention to diagnosis AHI, EHI, and chronic HIV infection among outpatients in a large Government Hospital in Malindi, coastal Kenya, who met the consensus risk score criteria. We report the yield of the HIV-testing intervention among individuals tested, and the time to ART initiation among individuals diagnosed.

Patients aged 18–35 years with unknown HIV status were screened to determine whether they met consensus risk score criteria [11]. Patients received a score of ‘1’ for young age (18–29 years), reported fever, fatigue, body pains, diarrhea, or sore throat, and a ‘3’ for reported genital ulcer disease. Patients with a risk score of at least 2 who agreed to the HIV-testing intervention were enrolled and received pretest counseling, after which a 4-ml blood sample was collected. Axillary temperature was measured in all patients, and documented fever defined as at least 37.5°C axillary. HIV testing was done in the presence of the patient using two rapid HIV antibody tests in parallel (Determine; Abbott Laboratories, Abbott Park, Illinois, USA; Unigold; Trinity Biotech plc, Bray, Ireland). Blood samples from participants whose rapid HIV antibody test results were HIV-negative or discordant were tested for HIV-1 RNA detection using the *Xpert HIV-1 Qual* assay (Cepheid, Sunnyvale, California, USA; prequalified by the WHO in 2016). Patients awaited Xpert results (∼1.5 h). All HIV-1-infected patients were offered immediate ART, in accordance with Kenyan guidelines [12].

From February to August 2016, 1063 (42.7%) of 2490 patients seeking care were screened for eligibility (Table 1). Approximately, one in three eligible patients refused research participation, and 706 patients enrolled in the study. Among patients with a documented refusal reason (54.4% of total refusals), the two most common reasons for refusing research participation were unwillingness to undergo HIV testing (41.1% in men, 30.3% in women; *P* = 0.15) and time constraints (17.9% in men, 21.8% in women; *P* = 0.5). Eligibility and enrolment did not differ by sex. The median age was 26 [interquartile range (IQR) 23–29) years in men and 25 (IQR 22–28) years in women. The median risk score was 3 (range 2–8) for men and 3 for women (range 2–7). Of the 706 participants, 74.1% had body pains, 70.7% fatigue, 50.1% reported fever, 24.5% sore throat, 11.6% diarrhea, and 8.5% ulcer disease (details in Table 1). In all, 107 (15.2%) patients had a documented fever.

The HIV-1 antibody prevalence was 3.9% [95% confidence interval (CI) 1.6–7.5%) in men and 3.2% (95% CI 1.8–5.1%) in women, and 24 seropositive patients were newly diagnosed (8 men and 16 women). Of 682 patients (197 men and 485 women) who had negative or discordant rapid test results, 6 (0.9%) had acute and early HIV infection (AEHI), including 3 women with AHI and 3 women with EHI. The median risk score for AEHI patients was 5 [range 3–7, statistically significantly different from non-AEHI patients (*P* = 0.029)]. Of the 6 AEHI patients, 6 (100.0%) had body pains, 4 (66.7%) fatigue, 4 (66.7%) reported fever, 3 (50.0%) sore throat, 3 (50.0%) ulcer disease, and none had diarrhea. Five of the 6 AEHI patients were under 30 years of age. None of the AEHI patients had a documented fever.

Of the 30 newly diagnosed HIV patients, 23 (76.7%; 6 men and 17 women) registered for care after a median of 0 days (range 0–52) and 21 (70.0%; 6 men and 15 women) started ART after a median of 4 days (range 0–71). Five (83.3%) of the six AEHI patients started ART after a median of 0 day (range 0–11). Tracing attempts were unsuccessful for the two patients who registered for care, but did not initiate ART. Among the seven patients who failed to register, one requested referral to another HIV care facility and six were lost to follow-up shortly after diagnosis.

The HIV-testing intervention in the present study was carried out in approximately 43% of patients seeking care. The addition of *Xpert HIV-1 Qual* testing led to an increase in confirmed diagnoses by 25% (from 24 to 30 cases); 10% were in women who would not otherwise have been diagnosed and 10% were in women with discordant test results, who may not have followed up with repeat testing as recommended. In targeted risk groups, such as patients presenting with sexually transmitted infections (STIs), AHI prevalence can be as high as 1.0%, as reported in STI clinics in Malawi [13]. That study led to a recommendation for universal HIV-1 RNA testing at STI clinics [13]. In our study, the prevalence of AEHI combined was 0.9%, suggesting that HIV-1 RNA testing among young general outpatients targeted by our risk score algorithm is a sensible strategy for programmatic implementation. Because the cost of HIV-1 RNA testing (US $14.90 per assay since July 2018) is considerable, there is growing consensus that detection of AEHI in resource-limited countries should be guided by algorithms that identify at-risk individuals [6].

Our study was conducted in a town with a high HIV-1 prevalence, but low HIV testing and ART coverage, at a hospital in which provider-initiated testing and counseling was not routinely offered to outpatients. In contrast with earlier AHI screening in 2013, we did not find AEHI among patients with a documented fever [2], diarrhea, or among men. That we did not find AEHI among men may simply be chance, or due to the lower numbers of men participating in this study (29.1% of the total), the lower HIV incidence among young men than young women in Kenya [14], or that men had higher refusal rates than women for HIV testing. Attrition was relatively high, with nine patients (30.0%) lost to follow-up before initiating ART. It is possible that some of these patients linked to care elsewhere without our knowledge, not wanting to be associated with our research facility, which is known to serve key populations. Our consensus AHI algorithm identified AEHI patients who were more ill than non-AEHI patients seeking urgent care. Further studies on the yield of our AHI testing algorithm, including evaluation the cost-effectiveness of this approach, will be conducted in other areas of coastal Kenya in an NIH-funded proof-of-concept trial among adult patients seeking urgent care (R01AI124968). Finding patients with AEHI will remain a much needed but elusive target for public health interventions while costs of AHI tests remain high. HIV-1 RNA testing is recommended for emergency room screening in resource-rich countries [1], but AHI diagnosis considered a ‘common occurrence’ overlooked in resource-constrained settings [15]. Our study showed that AEHI screening using a POC HIV-1 RNA assay is feasible, led to a substantial increase in confirmed HIV-1 diagnoses, and allowed the majority of the AEHI patients to start on immediate ART.

## Acknowledgements

We thank staff at the Malindi Subcounty Hospital and at KWTRP in Kilifi, Kenya. KWTRP is supported by core funding from the Wellcome Trust (#203077/Z/16/Z). This work was partially funded by the International AIDS Vaccine Initiative (IAVI) with the generous support of USAID and other donors; a full list of IAVI donors is available at www.iavi.org. EJS receives research funding from IAVI, NIH (grant R01AI124968), and the Wellcome Trust. SMG was supported by NIH (grant R01AI124968). This work was also supported through the Sub-Saharan African Network for TB/HIV Research Excellence (SANTHE), a DELTAS Africa Initiative [grant # DEL-15–006]. The contents are the responsibility of the study authors and do not necessarily reflect the views of USAID, the NIH, the United States Government, or the Wellcome Trust. This report was published with permission from KEMRI.

### Conflicts of interest

There are no conflicts of interest.

## Figures and Tables

**Table 1:** Screening outcomes of adult patients aged 18–35 years enrolled for acute HIV infection evaluation at outpatient care seeking, Kenya, 2016.

Outcomes	Men	Women	All
Screened	711	1779	2490
Eligible	318 (44.7% of screened)	745 (41.9% of screened)	1063 (42.7% of screened)
Enrolled	205 (64.5% of eligible)	501 (67.3% of eligible)	706 (66.4% of eligible)
Risk score, median (range)	3 (2–8)	3 (2–7)	3 (2–8)
Age, median (IQR)	26 (23–29)	25 (22–28)	25 (22–29)
Reported fever	116 (56.6%)	238 (47.5%)	354 (50.1%)
Fatigue	132 (64.4%)	367 (73.3%)	499 (70.7%)
Body pains	149 (72.7%)	374 (74.7%)	523 (74.1%)
Diarrhea	31 (15.1%)	51 (10.2%)	82 (11.6%)
Sore throat	57 (27.8%)	116 (23.2)	173 (24.5%)
Genital ulcer disease	15 (7.3%)	45 (9.0%)	60 (8.5%)
Chronic HIV^a^	8 (3.9%)	16 (3.2%)	24 (3.4%)
AHI^b^	–	3	3 (0.4%)
EHI^c^	–	3	3 (0.4%)
Started ART	6 (75.0%)	15 (68.0%)	21 (70.0%)

^a^Chronic HIV-seropositive on two rapid antibody tests.

^b^AHI: acute HIV infection (RNA-positive and anti-HIV antibodies undetectable).

^c^EHI: early HIV infection (RNA-positive and discordant HIV antibody test results).

## References

[1] White DAE Giordano TP Pasalar S Jacobson KR Glick NR Sha BE Acute HIV discovered during routine HIV screening with HIV antigen-antibody combination tests in 9 US emergency departments. *Ann Emerg Med* 2018; 72:29–40.e2. 29310870 10.1016/j.annemergmed.2017.11.027

[2] Sanders EJ Mugo P Prins HA Wahome E Thiong’o AN Mwashigadi G Acute HIV-1 infection is as common as malaria in young febrile adults seeking care in coastal Kenya. *AIDS* 2014; 28:1357–1363. 24556872 10.1097/QAD.0000000000000245 PMC4032215

[3] Rutstein SE Sellers CJ Ananworanich J Cohen MS The HIV treatment cascade in acutely infected people: informing global guidelines. *Curr Opin HIV AIDS* 2015; 10:395–402. 26371460 10.1097/COH.0000000000000193 PMC4739850

[4] World Health Organization. Consolidated guidelines on the use of antiretroviral drugs for treating and preventive HIV infection: recommendations for a public health approach. Geneva, 2nd ed.; 2016. 27466667

[5] DHHS. Panel on Antiretroviral Guidelines for Adults and Adolescents. Department of Health and Human Services. http://www.aidsinfo.nih.gov/ContentFiles/AdultandAdolescentGL.pdf [Accessed 30 April 2018]

[6] Rutstein SE Ananworanich J Fidler S Johnson C Sanders EJ Sued O Clinical and public health implications of acute and early HIV detection and treatment: a scoping review. *J Int AIDS Soc* 2017; 20:1–13. 10.7448/IAS.20.1.21579 PMC5515019 28691435

[7] Robb ML Eller LA Kibuuka H Rono K Maganga L Nitayaphan S Prospective study of acute HIV-1 infection in adults in East Africa and Thailand. *N Engl J Med* 2016; 374:2120–2130. 27192360 10.1056/NEJMoa1508952 PMC5111628

[8] Lindback S Karlsson AC Mittler J Blaxhult A Carlsson M Briheim G Viral dynamics in primary HIV-1 infection. Karolinska Institutet Primary HIV Infection Study Group. *AIDS* 2000; 14:2283–2291. 11089616 10.1097/00002030-200010200-00009

[9] Sanders EJ Price MA Karita E Kamali A Kilembe W Bekker LG Differences in acute retroviral syndrome by HIV-1 subtype in a multicentre cohort study in Africa. *AIDS* 2017; 31:2541–2546. 29028659 10.1097/QAD.0000000000001659 PMC5690309

[10] Sanders EJ Wahome E Mwangome M Thiong’o AN Okuku HS Price MA Most adults seek urgent healthcare when acquiring HIV-1 and are frequently treated for malaria in coastal Kenya. *AIDS* 2011; 25:1219–1224. 21505300 10.1097/QAD.0b013e3283474ed5

[11] Sanders EJ Wahome E Powers KA Werner L Fegan G Lavreys L Targeted screening of at-risk adults for acute HIV-1 infection in sub-Saharan Africa. *AIDS* 2015; 29 suppl 3:S221–S230. 26562811 10.1097/QAD.0000000000000924 PMC4714928

[12] Ministry of Health, National AIDS & STI Control Programme. Guidelines on use of Antiretroviral Drugs for Treating and Preventing HIV Infection in Kenya 2016. Nairobi, Kenya: NASCOP; 2016.

[13] Rutstein SE Pettifor AE Phiri S Kamanga G Hoffman IF Hosseinipour MC Incorporating acute HIV screening into routine HIV testing at sexually transmitted infection clinics, and HIV testing and counseling centers in Lilongwe, Malawi. *J Acquir Immune Defic Syndr* 2016; 71:272–280. 26428231 10.1097/QAI.0000000000000853 PMC4752378

[14] Borgdorff MW Kwaro D Obor D Otieno G Kamire V Odongo F HIV incidence in western Kenya during scale-up of antiretroviral therapy and voluntary medical male circumcision: a population-based cohort analysis. *Lancet HIV* 2018; 5:e241–e249. 29650451 10.1016/S2352-3018(18)30025-0

[15] Powers KA Cohen MS Acute HIV-1 infection in sub-Saharan Africa: a common occurrence overlooked. *AIDS* 2014; 28:1365–1367. 24959964 10.1097/QAD.0000000000000277

